# Is shrimp farming a successful adaptation to salinity intrusion? A geospatial associative analysis of poverty in the populous Ganges–Brahmaputra–Meghna Delta of Bangladesh

**DOI:** 10.1007/s11625-016-0356-6

**Published:** 2016-03-21

**Authors:** Fiifi Amoako Johnson, Craig W. Hutton, Duncan Hornby, Attila N. Lázár, Anirban Mukhopadhyay

**Affiliations:** 1 Department of Social Statistics and Demography & Centre for Global Health, Population, Poverty and Policy (GHP3), Faculty of Social, Human and Mathematical Sciences, University of Southampton, Southampton, United Kingdom; 2 GeoData Institute, Faculty of Social, Human and Mathematical Sciences, University of Southampton, Southampton, United Kingdom; 3 Faculty of Engineering and the Environment, University of Southampton, Southampton, United Kingdom; 4 School of Oceanographic Studies, Jadavpur University, Kolkata, India

**Keywords:** Poverty, Shrimp farming, Salinization, Ganges–Brahmaputra–Meghna delta, Bangladesh, Spatial analysis

## Abstract

The Ganges–Brahmaputra–Meghna delta of Bangladesh is one of the most populous deltas in the world, supporting as many as 140 million people. The delta is threatened by diverse environmental stressors including salinity intrusion, with adverse consequences for livelihood and health. Shrimp farming is recognised as one of the few economic adaptations to the impacts of the rapidly salinizing delta. Although salinity intrusion and shrimp farming are geographically co-located in the delta, there has been no systematic study to examine their geospatial associations with poverty. In this study, we use multiple data sources including Census, Landsat Satellite Imagery and soil salinity survey data to examine the extent of geospatial clustering of poverty within the delta and their associative relationships with salinity intensity and shrimp farming. The analysis was conducted at the union level, which is the lowest local government administrative unit in Bangladesh. The findings show a strong clustering of poverty in the delta, and whilst different intensities of salinization are significantly associated with increasing poverty, neither saline nor freshwater shrimp farming has a significant association with poverty. These findings suggest that whilst shrimp farming may produce economic growth, in its present form it has not been an effective adaptation for the poor and marginalised areas of the delta. The study demonstrates that there are a series of drivers of poverty in the delta, including salinization, water logging, wetland/mudflats, employment, education and access to roads, amongst others that are discernible spatially, indicating that poverty alleviation programmes in the delta require strengthening with area-specific targeted interventions.

**Electronic supplementary material:**

The online version of this article (doi:10.1007/s11625-016-0356-6) contains supplementary material, which is available to authorized users.

## Introduction

### Background

Although growth in shrimp farming in the Ganges–Brahmaputra–Meghna (GBM) delta of Bangladesh might be viewed as a useful adaptation to increasing salinity intrusion in the region (Primavera 1997; Paul and Vogl 2011; Belton et al. 2011; Kamruzzaman 2014), there are no systematic studies to establish the associations between shrimp farming, salinity intrusion and poverty, particularly for the vulnerable and marginalised population of the delta. The high demand and perceived monetary benefits of shrimp has inspired many farmers to convert farmlands intruded by saline water into shrimp farms, whilst others have actively encouraged saline water from marine sources into their farmlands to produce shrimp (Rahman et al. 2013). Cyclones and storm surges, particularly Cyclone *Sidr* in 2007 and *Aila* in 2009 have contributed to rapid salinization of the delta, including agricultural lands, freshwater ponds, canals and rivers (Mahmuduzzaman et al. 2014). This has been compounded by deforestation, particularly for large-scale shrimp farming, which has led to loss of protection from cyclones and storm surges. In addition, dam construction upstream reduces freshwater flow and increases sea water intrusion (Gain and Giupponi 2014; Mahmuduzzaman et al. 2014).

This study aims to examine union-level geospatial associations between shrimp farming, salinity intrusion and poverty in the delta. The specific objectives are to (1) use population level data to examine the extent of geographical variations in poverty in the delta, (2) identify the key drivers of poverty and (3) how the drivers of poverty are spatially distributed. The study hypothesises that whilst salinity intrusion is a major driver of poverty in the delta, the monetary benefits of shrimp farming adaptation is trivial to the marginalised and vulnerable local populations. In this study, the indicator of poverty is an asset index developed based on households ownership of assets and amenities, which several studies have associated with chronic poverty and the lack of human capital (Wietzke 2015; Stein and Horn 2012; Cooper and Bird 2012; Mackay and Lawson 2003). If the rapid growth in shrimp cultivation in the delta has had any measurable impact on the wellbeing of the local population, it should reflect in the levels and associations with poverty.

The first report of the Intergovernmental Panel on Climate Change recognised the effects of the changing climate and human induced activities on the environment and their subsequent impacts on the world’s ecosystems, which supports majority of the world’s poor (Melillo et al. 1990). In coastal regions of the world, increasing salinity, their impact on ecosystems and consequential effects on livelihoods of the poor, exacerbated by cyclones, sea level rise and storm surges are well documented in the research literature (Kotera et al. 2014; Shamsuddoha and Chowdry 2007). The poor in these regions are often compelled to adopt alternative livelihood strategies to cope with the adverse effects of environmental changes and stressors. The implications of the strategies adopted by the poor to cope with these environmental stressors and how these coping mechanisms enhance or aggravate their wellbeing and resilience have not received much research attention.

In the GBM delta of Bangladesh, salinity intrusion has adversely affected crop production, particularly rice which is a major livelihood and staple for the residents’ poor and marginalised populations (Haldar and Debnath 2014; Ali 2006). As a response to salinity intrusion in the delta, many farmers have adopted shrimp farming as an alternative livelihood source (Hossain et al. 2013; Rahman and Hossain 2009; Ali 2006; Mondal et al. 2001). As such the decline in livelihood from agricultural loss might be expected to be offset by increased activity in the saline shrimp sector.

Since the introduction of shrimp cultivation in the coastal belt of Bangladesh in the 1970s, the sector has grown tremendously and has become a very important sector to the economy. The saline coastal region of the country which was once dominated by rice farms is now eclipsed with shrimp ponds. It is estimated that there are 200,000 ha of coastal shrimp farms in Bangladesh, producing an average of 75,000 metric tonnes of shrimp per year and contributing 6 % of the country’s GDP (Rahman et al. 2013; Gammage et al. 2006). The growth in saline shrimp farming over the past 20 years can be viewed as an effective adaptation to increasing salinity in the region. However, the environmental impacts of this intense aquiculture practice (e.g. increasing soil toxicity) raise concerns over its sustainability. Intensive aquaculture has consequences for land tenure, livelihood displacements and income loss, food insecurity and health, rural unemployment, social unrest, conflicts and forced migration (Hossain et al. 2013; Swapan and Gavin 2011; Paul and Vogl 2011). Particularly, when there are concerns that the monetary benefits from shrimp are limited to only a few external investors  (Swapan and Gavin 2011). Although freshwater shrimp farming has less environmental impacts and higher yields compared to saline water shrimp farming, the practice of freshwater shrimp farming is limited because it is more capital intensive (Quassem et al. 2003).

In an environment that is progressively salinizing, matched against the need for adaptive approaches for sustainable production, there are concerns about the benefits of shrimp farming among the local population, particularly the poor and marginalised. In Bangladesh, it is estimated that net profit from shrimp cultivation is twelve times higher than high yielding rice varieties (Shang et al. 1998). However, due to low employment rates in shrimp farming and because many farmers do not have direct access to the international market, much of this profit is absorbed in a long supply chain of intermediaries (Ahsan 2011) as opposed to the local poor. Despite these concerns and increasing salinization of the delta region, understanding of the geographical impacts of shrimp farming by the poor to guide policy decisions and planning are lacking, whilst problems of ecosystem degradation and poverty continue to persist. In this study, we examine the extent of geospatial clustering of poverty in the GBM delta of Bangladesh, and the geospatial associative relationships with levels and intensities of soil salinity and shrimp farming as well as other possible environmental and socioeconomic drivers. Understanding the geospatial patterns in poverty and its geographical relationships with salinity and shrimp farming is vital for facilitating localised approaches for targeted interventions aimed at strengthening poverty reduction and environmental policies and programmes in the delta.

### Study setting

**Fig. 1:**
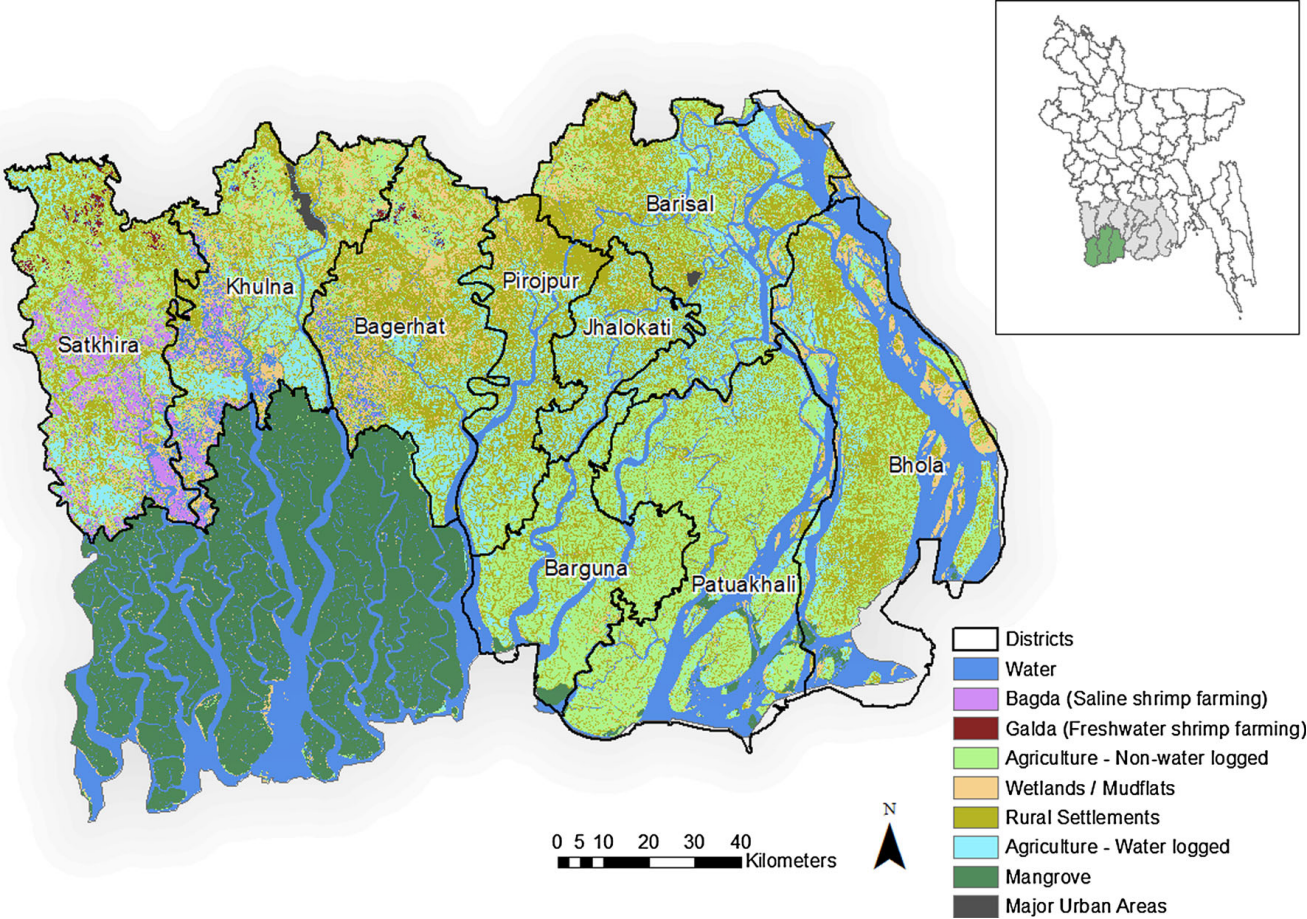
LandSat 5TM data imagery, *inset* map of Bangladesh highlighting the study area

The study focuses on the southcentral (Barisal, Bhola and Patuakhali districts) and southwestern (Bagerhat, Barguna, Jhalokati, Khulna, Pirojpur and Satkhira districts) coastal zones of the Bangladeshi GBM delta (Fig. 1). The GBM are trans-boundary rivers with a total area of 1.7 million square kilometres, covering Bangladesh (7 %), India (64 %), China (18 %), Nepal (9 %) and Bhutan (3 %) (FAO 2011). The study area covering 18,850 km^2^ is the tidal active part of the Bangladeshi delta of the GBM Rivers (Islam and Gnauck 2008; FAO 2011) to the east of the Padma river. The rivers and their tributaries dominate the regions’ environment and provide directly or indirectly the livelihoods and ecosystems services (water, soils, food and transport, amongst others) of many of its population (Hossain et al. 2015; Toufique and Turton 2002). Nonetheless, with an elevation of between 1 and 3 m above sea level, the rivers and their tributaries mediate most hazards in the region including floods and salinity intrusion (FAO 2011). Bangladesh being the lowest riparian country amongst all the GBM countries, suffers the most flood hazards during the monsoon rains (June to October), with catastrophic shocks, including morbidity and mortality, displacements and destruction to property (Doocy et al. 2013). The impacts are severe on agriculture and aquaculture (covering 45 and 11 % of the total area, respectively), the economic mainstay of the region (Doocy et al. 2013). Dry season low flows, exacerbated by upstream flow diversions causes salinization of rivers and groundwater resources, thus increasing soil salinity (Gain and Giupponi 2014; Mirza 1998).

Although the delta provides a range of important ecosystems which make the region highly suitable for agriculture, poverty levels remain high with more than one-half of the population living on less US$1.25 per day (WRI 2005). With a population of 14 million, and a high population density of 750 people per square kilometre, poverty in the region is aggravated by landlessness (more than one-half of rural households do not own land) and salinity intrusion which threatens ecosystems and agricultural production (Gain et al. 2008; Islam and Gnauck 2008; Toufique and Turton 2002). Although many agricultural lands in the region have been converted into shrimp farms due to salinity intrusion, there is no established scientific intelligence whether they help alleviate poverty amongst vulnerable and marginalised populations in the region.

To examine the geospatial variations in poverty and their associated factors, the analysis was conducted at the union level, which is the lowest local government administrative unit in Bangladesh. The country operates a four-tier local government system, consisting of Divisions (7), Districts or Zilas (64), Sub-districts or Upazilas (492) and Unions (4501) (MoHFW 2012; Panday 2011). A union consists of nine Wards or Villages, and is governed by a Union Council that is primarily responsible for agricultural, industrial and community development. The study covered the 653 unions in 70 Upazilas in the central and western coastal zones of the Bangladesh delta of the GBM Rivers. The 2011 Bangladesh Population and Housing Census classified unions into rural and urban (cities, municipalities and Upazila headquarters). The study area was classified into 497 rural and 156 urban unions. Four of the nine districts in the study area (Bagerhat, Satkhira, Pirojpur and Khulna) are classified amongst the major shrimp-producing districts in Bangladesh (FAO 2015).

## Data

The data for the analysis come from the 2011 Bangladesh Population and Housing Census (BPHC), 2009 Bangladesh Soil Salinity Survey (BSSS) and 2010 Bangladesh Landsat 5TM, supplemented with the 2010 MODIS Terra Satellite Imagery (MODIS TSI) of Bangladesh. The 2011 BPHC is the fifth post-independence census undertaken in Bangladesh, conducted by the Bangladesh Bureau of Statistics from 15 to 19 March 2011 (BBS et al. 2012). The outcome variable of interest, ‘asset poverty’ was derived from the BPHC, whilst the primary factors, percentage of union area affected by different intensities of salinity and percentage of union area used for saline and freshwater shrimp farming were derived from the BSSS and Landsat, respectively. The environmental and socioeconomic controls were derived from the BPHC, Landsat and MODIS TSI. Table 1 shows the variables selected for the analysis, the source, definition, categorisation and coding of categorical variables. Data from the 2011 Bangladesh Demographic and Health Survey (BDHS) were used to validate the robustness of the outcome variable derived from the Census data. The calibration of the data and variables is discussed in the subsequent sections.

**Table 1:** Dependent and independent variables

Variables and categorisation	Definition	Year and source	Type	Categorical variable coding
Dependent variable				
Asset poverty	Multidimensional score derived based on ownership of assets and amenities using maximum likelihood factor analysis. The scores were aggregated into quintiles	2011 BPHC	Categorical	1 = bottom quintile, 2 = second quintile, 3 = middle quintile, 4 = fourth quintile, 5 = top quintile
Independent variables				
*Administrative level controls*				
Division	The highest local government administrative unit	2011 BPHC	Categorical	0 = Barisal, 1 = Khulna
Type of union	2011 BPHC classification of unions based on amenities within each union	2011 BPHC	Categorical	0 = urban, 1 = rural
*Primary factors*				
Soil salinity				
2–4 dS/m salinity	% of union area affected by low salinity (2–4 dS/m)	2009 BSSS	Continuous	
4.1–8 dS/m salinity	% of union area affected by moderate salinity (4.1–8 dS/m)	2009 BSSS	Continuous	
8.1–12 dS/m salinity	% of union area affected by high salinity (8.1–12 dS/m)	2009 BSSS	Continuous	
>12 dS/m salinity	% of union area affected by very high salinity (>12 dS/m)	2009 BSSS	Continuous	
Shrimp farming				
Saline water shrimp farming	% of union area use for saline water shrimp farming	2010 Landsat 5TM	Categorical	0 = none, 1 = low (less than 1 %), 2 = moderate (1–10 %), 3 = high (greater than 10 %)
Fresh water shrimp farming	% of union area used for fresh water shrimp farming	2010 Landsat 5TM	Categorical	0 = none, 1 = low (less than 1 %), 2 = high (greater than 1 %)
*Environmental controls*				
Mangrove forest	Presence of mangrove forest within union	2010 Landsat 5TM/MODIS TSI	Categorical	0 = no mangrove, 1 = mangrove
Water logged agricultural	% of water logged agricultural lands in a union	2010 Landsat 5TM/MODIS TSI	Continuous	
Permanent open water bodies	% of union area made up of permanent open water bodies	2010 Landsat 5TM/MODIS TSI	Continuous	
Wetland and mudflats	% of union area made up of wetland and mudflats	2010 Landsat 5TM/MODIS TSI	Continuous	
*Socioeconomic controls*				
Employment	Ratio of persons aged 15–60 years working and the population aged 15–60 (percentage)	2011 BPHC	Continuous	
Literacy	Ratio of persons aged 7+ years that are able to write a simple letter and the population aged 7+ years (percentage)	2011 BPHC	Continuous	
School attendance	Ratio of pupils aged 6–14 years registered or enrolled in school and the population age 6–14 years (percentage)	2011 BPHC	Continuous	
Population density	Number of people per square kilometre of area	2011 BPHC	Continuous	
Dependency ratio (%)	Ratio of the population aged 0–14 years and 60+ years to the population aged 15–59 years	2011 BPHC	Continuous	
Average household size	Number of persons living in a household, where a household is a group of persons, related or unrelated, living together and taking food from the same kitchen	2011 BPHC	Continuous	
Major roads density	Ratio of the total length of all major (national, regional and district) roads to union area, expressed as kilometre of road per kilometre square of area	2011 BDRH	Continuous	

*BPHC* Bangladesh Population and Housing Census, *BSSS* Bangladesh Soil Salinity Survey, *MODIS TSI* MODIS Terra Satellite Imagery, *BDRH* Bangladesh Department of Roads and Highways

### Dependent variable

The dependent variable representing the broad domain of poverty is ‘asset poverty’, derived as a score based on households’ ownership of assets and amenities. Data constraint remains a major limitation in examining geospatial variations in poverty. Conventional approaches for quantifying poverty levels have focused on measures such as the poverty headcount, income share and the poverty gap, amongst others (Haughton and Khander 2009). These measures require information on income, expenditure and or consumption. However, for most low- and middle-income countries these data are often unavailable or unreliable (Meyer and Sullivan 2003; Nicoletti et al. 2011); and even where they are available, they cannot be used to examine poverty at lower levels of geographical aggregation such as unions or districts. For example, population surveys such as the Living Standards Surveys, Multiple Indicator Cluster Surveys and Household Income and Expenditure Surveys collect substantial amount of nationally representative data and often cover information on income, expenditure and consumption. However, they cannot be used to derive estimates at lower levels of aggregation because of small sample sizes which lead to high levels of sampling variability and inadequate precision (Pfeffermann 2002). Censuses on the other hand are representative at the lowest geospatial disaggregation, but they do not often collect information on income, expenditure or consumption.

To overcome the limitations of the conventional approaches, studies have used alternative techniques based on households’ ownership of assets and amenities (Filmer and Prittchet 2001). Research evidence shows that asset poverty robustly captures the multidimensionality of poverty (Filmer and Prittchet 2001) and is often associated with chronic poverty and lack of human capital (Wietzke 2015; Stein and Horn 2012; Cooper and Bird 2012; Mackay and Lawson 2003). Conversely, household expenditure data do not capture longer term poverty trends, whilst consumption data are often affected by seasonal changes and economic shocks (Sahn and Stifel 2003).

A key advantage of the asset approach is that most censuses collect information on households’ ownership of asset and amenities which can be used to examine variations in poverty at the lowest geographic unit. In this study, the assets and amenities data derived from the 2011 BPHC were housing structure (pucka, semi pucka, kutcha and jhupri), sources of drinking water (tap, tube well and others), type of toilet facility (water sealed, non-water sealed, non-sanitary and no toilet) and electricity connectivity. With regards to housing structure, pucka refers to houses built with permanent materials such as burnt bricks or concrete, whilst kutcha are those built with nondurable materials such mud floors and metal sheet roofs and/or walls (Bern et al. 1994; Nenova 2010; GFDRR et al. 2014). Semi-pucka is a hybrid of pucka and kutcha, where floors and/or walls are bricks or concrete but the rest are made of metal sheets (Nenova 2010; GFDRR et al. 2014). Very poor quality houses such as those with earthen floors and mud, bamboo or straw walls or roofs are referred to as jhupri (Bern et al. 1994; GFDRR et al. 2014). A unions’ asset poverty status was measured as a multidimensional score based on the percentage of households in a union owing these assets and amenities. The computation procedures are described in the methods section.

A major limitation of using Census data for constructing an asset index is that it collects very limited information on ownership of assets and amenities when compared to household surveys such as the Demographic and Health Survey (DHS) and Living Standards Survey (LSS). The 2011 BPHC collected information only on housing structure, sources of drinking water, type of toilet facility and electricity connectivity, whilst the DHS collects all the indicators mentioned plus ownership of vehicle, television, radio, telephone, electric fan, water pump, autobike, rickshaw, bicycle, motorcycle, scooter and refrigerator amongst others (Rutstein and Johnson 2004). In this regard, validation of the Census asset index in relation to the DHS is important.

### Independent variables

The independent variables selected for the analyses are classified into primary factors (levels and intensities of salinization and type of shrimp farming), and environmental and socioeconomic controls. We also accounted for the division (Barisal versus Kulhna) in which the union is located and whether the union is classified as urban or rural.

#### Primary factors

The soil salinity data were derived from the 2009 BSSS (Ahsan 2012), whilst union area used for shrimp farming was extracted from the Landsat 5TM remotes sensing images. The BSSS data were intersected with the union layer map to extract the union area intruded by salinity. The BSSS classified soil salinity into four intensities: (i) low salinity (2–4 dS/m), (ii) moderate salinity (4.1–8 dS/m), (iii) high salinity (8.1–12 dS/m) and (iv) very high salinity (12 dS/m or higher). We calculated the amount of union area affected by each of the soil salinity intensities. The classification of salinity intensities is primarily based on their constraints to agricultural productivity (Ahsan 2012).

From the Landsat 5TM imagery we calculated (see ESM Appendix I for more information on the extraction procedure and accuracy assessment) the percentage of union area used for saline and freshwater shrimp farming. Almost three-fourth of unions did not practise shrimp farming; therefore, the percentage of union area used for saline and freshwater shrimp farms was recoded into categorical variables to avoid the problem of zero-inflated covariates (Bagozzi et al. 2014). A careful consideration was given to ensure that the number of unions in each category was large enough to achieve model convergence and robust model parameter estimates. The percentage of union area used for saline water shrimp farming was classified into four categories: (i) no saline water shrimp farming, (ii) low saline water shrimp farming (less than 1 % of union area), (iii) moderate saline water shrimp farming (1–10 % of union area), and (iv) high saline water shrimp farming (greater than 10 % of union area). The percentage of union area used for freshwater shrimp farming was categorised into unions with (i) no freshwater shrimp farming, (ii) low freshwater shrimp farming (less than 1 % of union area), and (iii) high freshwater shrimp farming (greater than 1 % of union area). Union area used for freshwater shrimp farming was categorised into three groups because the practice is very limited in the study area and there are only eight unions where more than 10 % of the union area is used for freshwater shrimp farming.

#### Environmental and socioeconomic controls

The environmental and socioeconomic predictors were selected based on the literature and data availability. Merrick (2002) in a global context discussed the relationship between fertility, household size and poverty. Islam and Chuenpagdee (2013) analysed the determinants of poverty in the Bangladesh Sundarbans and found that employment, family size, dependency ratio, land ownership and access to information are important predictors of poverty. Khudri and Chowdhury (2013) analysed the 2007 BDHS and reported that administrative region, rural–urban residence, ownership of agricultural land, educational background and employment status are key predictors of poverty. Khandker et al. (2009) analysed the importance of access to road and poverty alleviation in Bangladesh. Studies that have analysed the relationship between poverty and environmental covariates include Hussain et al. (2006) and Rabbani et al. (2013). Hussain et al. (2006) reported a significant association between poverty and agricultural productivity, water distribution, land holding and also family size. Rabbani et al. (2013) elucidated the relationship between poverty and access to water, dependency on wetlands, salinity intrusion and cyclones in coastal areas of Bangladesh.

Based on literature and data availability, the environmental controls extracted from the Landsat 5TM are the percentage of agricultural lands in a union that is water logged, percentage of union area that was mangrove forest and permanent open water bodies. 95 % of the unions in the study area do not have mangrove forest; therefore, the data were categorised into unions with (i) mangrove forest and (ii) those without mangrove forest, to avoid the problem of zero-inflated covariates. Validation of the land cover and land use map. The data extraction procedures are discussed in ESM Appendix I. A comprehensive accuracy assessment of the Landsat 5TM imagery-based land cover and land use map was conducted using photo interpretation of Google Earth imagery from the same period of analysis. The results showed that 80 % of the classes were correctly classified, with a Kappa statistics of 76 %, implying almost a perfect agreement between what was observed and what was expected (see ESM Appendix I for further details).

The socioeconomic controls were derived from the 2011 BPHS. The Census provided information on population distribution and dynamics (births, deaths, migration and marital status) as well as data on socioeconomic conditions (education, literacy, economic activity and employment, religion, ethnicity and disability) within households. The socioeconomic controls include the percentage of 15–64-year-olds who are employed, population aged 15 years or older who are literate, children aged 6–14 years who are in school, population density, dependency ratio and average household size. A spatial data layer of national road network from a national programme of land surveillance conducted by the Bangladesh Department of Roads and Highways (BDRH) was used to calculate the major road density within each union. All the socioeconomic controls were analysed as continuous covariates.

## Methods

### Derivation of asset poverty scores

A multidimensional matrix of indicators on housing structure, type of toilet facility, source of drinking water and electricity connectivity were used to construct an asset poverty score at the union level (Filmer and Pritchet 2001). A maximum likelihood factor analysis technique was used to derive the score (Filmer and Pritchet 2001; Rutstein and Johnson 2004). The motivation for using maximum likelihood factor analysis is that it circumvents the problem of multicollinearity and assigns indicator weights based on the variations in ownership of assets and amenities (Jones and Andrey 2007). Factor analysis assigns higher weights (factor scores) to assets and amenities that are more inequitably distributed between households than those that are homogeneously owned, thereby capturing inequality between households (McKenzie 2005; Vyas and Kumaranayake 2006). Generally, variables with higher factor scores are associated with high wealth status, whilst low scores are associated with low wealth status. For example, if ownership of a pucka house is assigned a higher factor score in comparison to ownership of a jhupri house, then households with pucka house are considered to have higher socioeconomic status than those with jhupri house. The implication being that ownership of quality housing being more strongly correlated with variables expected to be associated with high socioeconomic status. The reader is referred to Rutstein and Johnson (2004) and Vyas and Kumaranayake (2006) for more detailed discussion on the construction of the indices.

### Validation of asset poverty scores

Data on households’ wealth index from the 2011 BDHS were used to validate the asset poverty scores derived from the Census. The BDHS is a nationally representative cross-sectional survey that collects information on ownership of a wider range of assets and amenities when compared to the Census; however, samples sizes within unions are not large enough for inference at that level. Since the Census collects only limited information on ownership of assets and amenities it is important to validate the robustness of using these data to examine variations in socioeconomic status. Validation of the asset poverty scores was performed by calibrating the scores computed by the National Institute of Population Research and Training et al. (2013) at the Upazila level and comparing them with the corresponding union-level estimates derived from the Census.

### Statistical analysis

The join-count spatial autocorrelation technique was used to examine whether spatial patterns of asset poverty amongst the unions in the study area are significantly random or clustered (Cliff and Ord 1981). Bayesian Geo-additive Semi-parametric (BGS) regression was used to examine the spatial differentials in asset poverty at the union level and the extent to which the primary and control factors explain the observed spatial differentials (Brezger et al. 2005). A key advantage of BGS techniques are that they allow unobserved spatial heterogeneity (both spatially structured and unstructured) to be accounted for. BSG techniques also allow for simultaneous estimation of non-linear effects of continuous covariates as well as fixed effects of categorical and continuous covariates in addition to spatial effects. Details of the statistical formulation of the BGS model are provided in ESM Appendix II.

A sequential model building approach was adapted to examine how the primary and control factors help explain the spatial variations in asset poverty across the delta. A base model (Model 1) accounting for the divisional and rural–urban classification of the unions was first fitted to account for the effect of administrative structure of the delta. Model 2 included the spatial effects to examine if there is significant spatial clustering in asset poverty across the delta. In Model 3, the primary factors were included in the model to examine how much of the spatial differentials in asset poverty is due to the primary factors. The environmental and socioeconomic controls were then included in Models 4 and 5, respectively, to ascertain their effects and also the independent effect of the primary factors. In both Models 4 and 5, all continuous variables were fitted as non-linear effects. However, to attain a parsimonious model, continuous covariates which exhibited linear effects in Models 4 and 5 were fitted as fixed effects in Model 6 (final model). Only covariates significant at *p* < 0.05 are retained in the model, except for the primary factors which are the principal covariates addressing the research question (Snijders and Bosker 2012). The analysis is performed using the BayesX statistical software (Brezger et al. 2005).

The Akaike Information Criterion (AIC) was used to identify the best fitted model. The AIC was the preferred model selection criteria because it accounts for spatial correlation in the selection of variables (Hoeting et al. 2006; Lee and Ghosh 2009). The computed AIC for each of the models (changes in the AIC) are compared and the model with the smallest AIC is selected, demonstrating that the model with the smallest AIC is the model that is closest to the true model (Lee and Ghosh 2009). Since there are no internationally accepted tables to ascertain how large the difference in AIC between models should be to indicate a best fit, it is difficult to judge how much statistical importance should be attached to a difference in AIC between candidate models. In other words, the difference in AIC does not indicate the weight of evidence in favour of model over others (Wagenmakers and Farrell 2004). In this study, the Akaike weight was used to assess importance of evidence in favour of the best model. The Akaike weight *w*_*r*_ for model *r* is expressed as

$$w_{r} = \frac{{{ \exp }\left\{ { - \frac{1}{2}\Delta_{j} ({\text{AIC}})} \right\}}}{{\mathop \sum \nolimits_{r = 1}^{R} { \exp }\left\{ { - \frac{1}{2}\Delta_{r} ({\text{AIC}})} \right\}}}$$

where $\Delta_{j} ({\text{AIC}})$ is the difference between the model with the lowest AIC and the AIC for each of the other models and *R* is the number of fitted models. Akaike weight ranges between 0 and 1, with the sum of all candidate models equal to 1, and analogous to the probability that model *R*_*r*_ is the best model given the available data and all candidate models (Wagenmakers and Farrell 2004). The strength of evidence in favour of one model over the other is determined by dividing their Akaike weights.

## Results

The first factor loading from the maximum likelihood factor analysis accounted for 33 % of the variability in ownership and quality of assets and amenities. The first factor loadings (housing structure: pucka = 0.87, semi-pucka = 0.78, Kutcha = −0.86, Jhupri = −0.31; type of toilet facility: water sealed = 0.52, non-water sealed = −0.20, non-sanitary = −0.37, no toilet = −0.43; source of drinking water: tap = 0.39, tube well = 0.23 and other = −0.36; electricity connectivity = 0.90) clearly indicate that unions which scored high on the first factor were those where a higher percentage of households own high-quality assets and amenities. The first factor score was, therefore, selected to represent the unions’ asset poverty score (Filmer and Pritchet 2001). Although, the first factor loading explained only 33 % of the variability in the data, we avoided combing multiple factors because research evidence has shown that only the first factor score is necessary for measuring wealth differentials (Filmer and Pritchett 2001; McKenzie 2005; Vyas and Kumaranayake 2006). A systematic analysis of combing multiple factors has shown that this distorts what the factors capture and their meaning may be lost making them difficult to interpret (McKenzie 2005). The first factor score was categorised into quintiles and mapped to show the extent of spatial clustering in asset poverty.

**Fig. 2:**
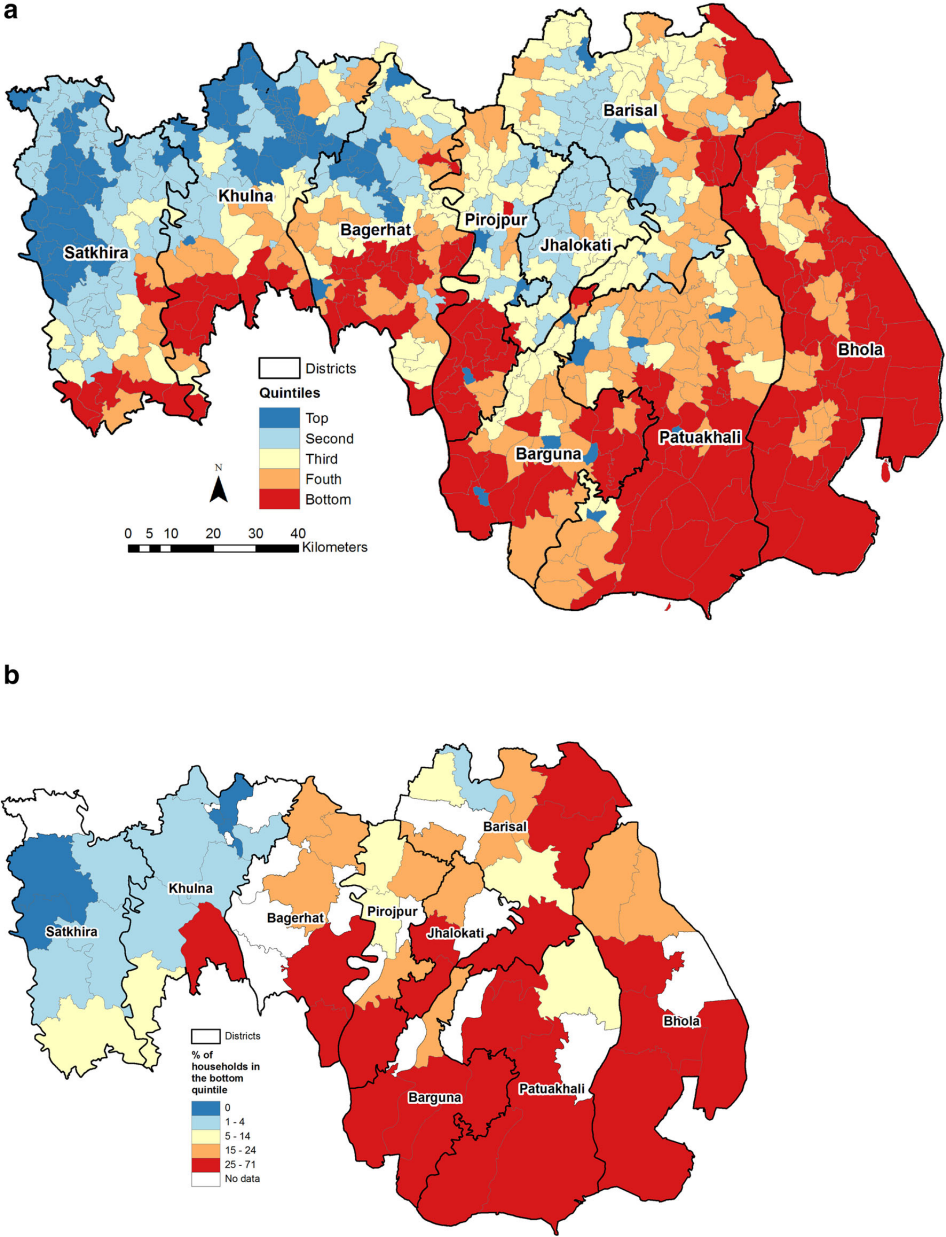
Observed **a** union-level (census-based) and **b** upazila-level (BDHS-based) geospatial variations in asset poverty in the Ganges–Brahmaputra–Meghna delta of Bangladesh

The extent of geospatial variations in asset poverty in the delta is shown in Fig. 2a. The figure shows the first factor score aggregated into quintiles. To validate the results presented in Fig. 2a, the percentage of households in the bottom quintile of the asset wealth score in each Upazila derived from the 2011 BDHS are shown in Fig. 2b. A comparison of Fig. 2a and 2b shows a clear semblance, suggesting that Fig. 2a captures robustly the union-level geospatial differentials in asset poverty. Figure 2a reveals strong clustering of asset poverty in the delta. The poorest unions are concentrated in the Bhola district and the unions close to the Sundarbans. In the Bhola district, more than one-half (58.2 %) of all the unions are in the bottom quintile. The Pirojpur district recorded the second highest percentage (34.9 %) of unions in the bottom quintile. About one-fourth of all unions in the Barguna (25.9 %), Patuakhali (25.6 %) and Bagerhat (25.0 %) districts are also in the bottom quintile. Asset poverty is lowest in the unions in Jhalokati (2.9 %), Barisal (5.7 %), Satkhira (9.5) and Khulna (9.7) districts where less than one-tenth of unions in those districts are in the bottom quintile.

To examine the spatial correlates of the selected covariates with asset poverty, the multivariate analysis focused on the bottom twenty percent (i.e. bottom quintile). This is consistent with distribution of chronic poverty in Bangladesh, where 19.5 % of the population suffers from extreme poverty (IMF 2012). A spatial autocorrelation analysis using the joint count approach revealed that unions in the bottom quintile are 2.94 times more likely to be neighbours than would be expected under a random spatial pattern (*Z*[BW] = −18.87, *p* < 0.05). This finding shows that the poorest unions are more concentrated in some parts of the study area when compared to others.

Table 2 shows the estimated posterior odds ratios and their corresponding 95 % credible intervals for the effects of fixed covariates on poverty along with their estimated AIC. The results show that when the spatial effects were included in the model (Model 2) the AIC decreased by 185.02 when compared to Model 1 which accounts for only the administrative level factors. The large decline in the AIC indicates that the spatial effects are required in the model. The spatial effects are a proxy for unaccounted spatially correlated covariate information, indicating that after accounting for the administrative effects, there exist significant geospatial clustering in asset poverty in the delta. The primary factors were then included in the model (Model 3) to examine their geospatial associations with asset poverty. The result presented in Table 2 shows that the AIC reduced further by 6.44. The relatively small decline in the AIC suggests that not all the primary factors may be spatially correlated with asset poverty. To identify the independent effect of the primary variables on poverty, the environmental and socioeconomic controls were included in the model (Models 4 and 5, respectively). When the environmental and socioeconomic controls were included the model the AIC reduced by 43.87 and 57.30, respectively, indicating that the controls have important associations with the spatial clustering of asset poverty in the delta. Using a flexible non-parametric modelling approach (Models 2–5) we were able to detect continuous variables with linear and those with non-linear effects. All continuous variables that exhibited linear associations were fitted as fixed effects in Model 6. The Akaike weights presented in Table 2 show that Model 6 is the best candidate model and is 2.62 times more likely to be the best model when compared to Model 5.

**Table 2:** Posterior odds ratios of the fixed effects and their corresponding 95 % credible intervals

Variables	Model 1, OR (95 % CI)	Model 2, OR (95 % CI)	Model 3 OR (95 % CI)	Model 4	Model 5, OR (95 % CI)	Model 6, OR (95 % CI)
Location effect						
Division						
Barisal	1.00	1.00	1.00	1.00	1.00	1.00
Khulna	0.42 (0.28, 0.65)**	2.15 (0.22, 21.07)	2.18 (0.22, 21.24)	1.28 (0.12, 13.2)	0.73 (0.06, 8.42)	0.71 (0.07, 7.8)
Type of union						
Urban	1.00	1.00	1.00	1.00	1.00	1.00
Rural	4.41 (2.34, 8.31)**	3.28 (1.12, 9.66)*	3.03 (1.03, 8.91)	2.85 (0.91, 8.92)	1.89 (0.51, 7.00)	1.89 (0.52, 6.88)
Primary variable						
% union area saline with						
2–4 dS/m salinity			Non-linear	Non-linear	Non-linear	1.01 (0.98, 1.04)
4.1–8 dS/m salinity			Non-linear	Non-linear	Non-linear	1.04 (1.00, 1.07)*
8.1–12 dS/m salinity			Non-linear	Non-linear	Non-linear	1.04 (1.00, 1.08)*
>12 dS/m salinity			Non-linear	Non-linear	Non-linear	1.07 (1.00, 1.14)*
Union area for SWS farming						
None			1.00	1.00	1.00	1.00
Low (less than 1 %)			1.20 (0.37, 3.86)	1.19 (0.34, 4.15)	1.37 (0.35, 5.32)	1.36 (0.36, 5.14)
Moderate (1–10 %)			1.88 (0.25, 13.94)	1.08 (0.10, 11.34)	1.78 (0.13, 24.57)	1.79 (0.14, 23.49)
High (greater than 10 %)			1.39 (0.10, 20.08)	0.47 (0.02, 11.14)	0.30 (0.01, 9.42)	0.30 (0.01, 8.85)
Union area for FWS farming						
None			1.00	1.00	1.00	1.00
Low (less than 1 %)			0.81 (0.26, 2.47)	0.58 (0.17, 2.01)	0.67 (0.16, 2.79)	0.66 (0.16, 2.70)
High (greater than 1 %)			0.70 (0.06, 8.11)	0.91 (0.05, 15.01)	0.42 (0.01, 16.86)	0.41 (0.01, 16.22)
Environmental controls						
Mangrove						
Unions with no mangrove				1.00	1.00	1.00
Unions with mangrove				7.85 (1.46, 42.28)*	6.68 (1.14, 39.10)*	6.05 (1.35, 27.16)*
Water logged agricultural				Non-linear	Non-linear	1.02 (1.00, 1.05)*
Permanent open water bodies				Non-linear	Non-linear	1.03 (1.00, 1.06)*
Wetland and mudflats				Non-linear	Non-linear	Non-linear
Socioeconomic controls						
% 15–64 years employed					Non-linear	Non-linear
% 15 years or older who are literate					Non-linear	0.90 (0.83, 0.98)*
% 6–14-year-olds in school					Non-linear	0.84 (0.73, 0.98)*
Major roads density within union					Non-linear	0.01 (0.00, 0.45)*
−2 log-likelihood	613.10	172.38	176.77	153.14	127.59	131.40
AIC	619.10	434.08	427.64	383.77	326.47	324.54
Change in AIC	–	185.02	6.44	43.87	57.3	1.93
Akaike weight	0.00	0.00	0.00	0.00	0.28	0.72

Model 1: locational effects only, Model 2: locational effects + structured spatial effects, Model 3: locational effects + primary factors + structured spatial effects, Model 4: locational effects + primary factors + environment controls + structured spatial effects, Model 5: locational effects + primary factors + environment controls + socioeconomic controls + structured spatial effects, Model 6: all non-linear effects fitted as fixed effects

*SWS* saline water shrimp, *FWS* freshwater shrimp

** *p* < 0.01, * *p* < 0.05

Model 6 shows a significant association between asset poverty and percentage of union area inundated by different intensities of salinization, even after accounting for the administrative effects and control variables (Table 2). However, the percentage of union area used for both saline and freshwater shrimp farming are not significantly associated with asset poverty. The estimated posterior odds ratios show that percentage of union area affected by low (2–4 dS/m) salinity does not significantly influence poverty. However, a percentage increase in union area affected by moderate (4.1–8 dS/m) and high (8.1–12 dS/m) salinity, both increases the odds of a union being in the bottom quintile by four percent. For high salinity intensity of 12 dS/m or higher, a percentage increase in union affected, increases the odds of being in the bottom quintile by seven percent. This suggests that increase in levels and intensities of salinity in a union increases the probability of the union being poor.

**Fig. 3:**
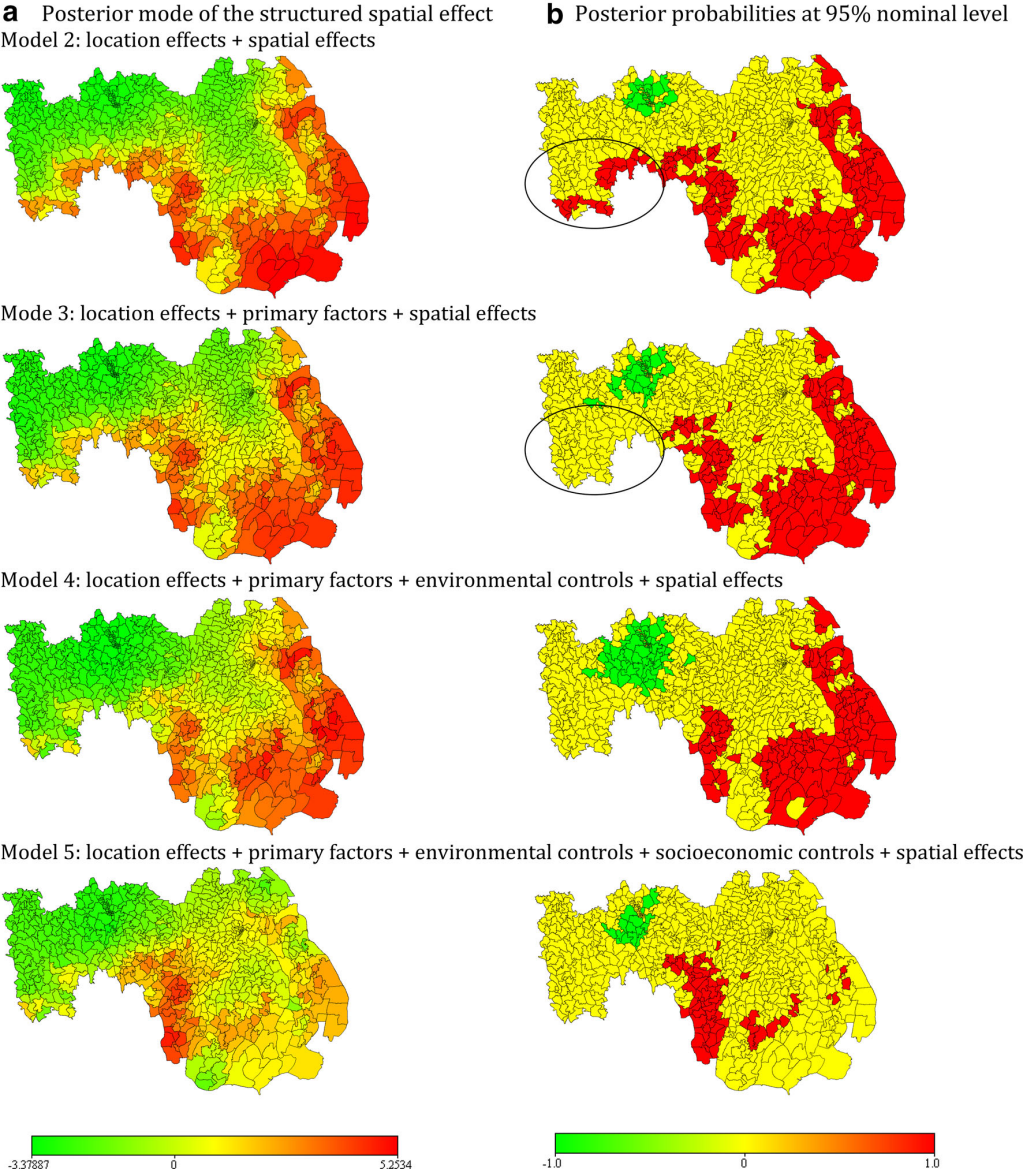
**a** Posterior mode of the structured spatial effects and **b** corresponding posterior probabilities at 95 % nominal. The posterior mode of the structured spatial effects show unions where asset poverty is high (*red*), low (*green*) and where the probability of being poor is not markedly different from not being poor (*yellow*), adjusting for the variables in the model. The posterior probabilities at 95 % nominal level show unions with *statistically significantly* high (*red*) asset poverty (95 % credible intervals lie in the *positive*), low (*green*) (95 % credible intervals lie in the *negative*) and (*yellow*) where they are not statistically significant (95 % credible intervals include 0). The posterior probabilities are used to identify spatial correlations of the covariates with poverty by comparing colour changes (*red* to *yellow* or *green* to *yellow*) between models. For example, **b** shows that for unions in close proximity to the Sundarban (*highlighted*) the posterior probabilities were significant for Model 2 but became statistically insignificant when the primary factors were included in the model (Model 3), indicating that the primary factors are significantly associated with asset poverty in those unions. In addition, a *cluster of similar colours* indicate statistical dependence in asset poverty

The posterior mode of the structured spatial effects (Fig. 3a) and their corresponding posterior probabilities at the 95 % nominal level (Fig. 3b) are used to examine the spatial drivers of poverty in the delta. The posterior mode of the structured spatial effects shows unions where asset poverty is high (red), low (green) and where they are trivial (yellow). The posterior probabilities at the 95 % nominal level show unions where asset poverty are statistically significantly high (red), significantly low (green) and where the effects are not significant (yellow). Where the posterior probabilities do not show statistically significant effects (yellow), the odds of a union being in the bottom quintile are not significantly different from the odds of being in the other quintiles. The spatial effects are a proxy for unaccounted spatially correlated covariate information. Therefore, using a sequential modelling approach, we were able to detect covariates that were spatially correlated with poverty in the delta. The posterior probabilities are used to identify the spatial correlations of the covariates with poverty by comparing colour changes (red to yellow or green to yellow) between models (Fig. 3b), i.e. examining where the estimated posterior mode of the structured spatial effects (Fig. 3a) becomes statistically non-significant (Fig. 3b) after covariates are added to the model.

In Fig. 3a, Model 2 shows that after accounting for the administrative effects, asset poverty remained significantly high in most unions in the Bhola and Patuakhali districts, as well as the unions in close proximity to the Sundarban. To identify unions where the primary factors are significantly associated with asset poverty, we compared the estimated posterior probabilities at 95 % nominal level from Models 2 and 3 (Fig. 3b).

**Fig. 4:**
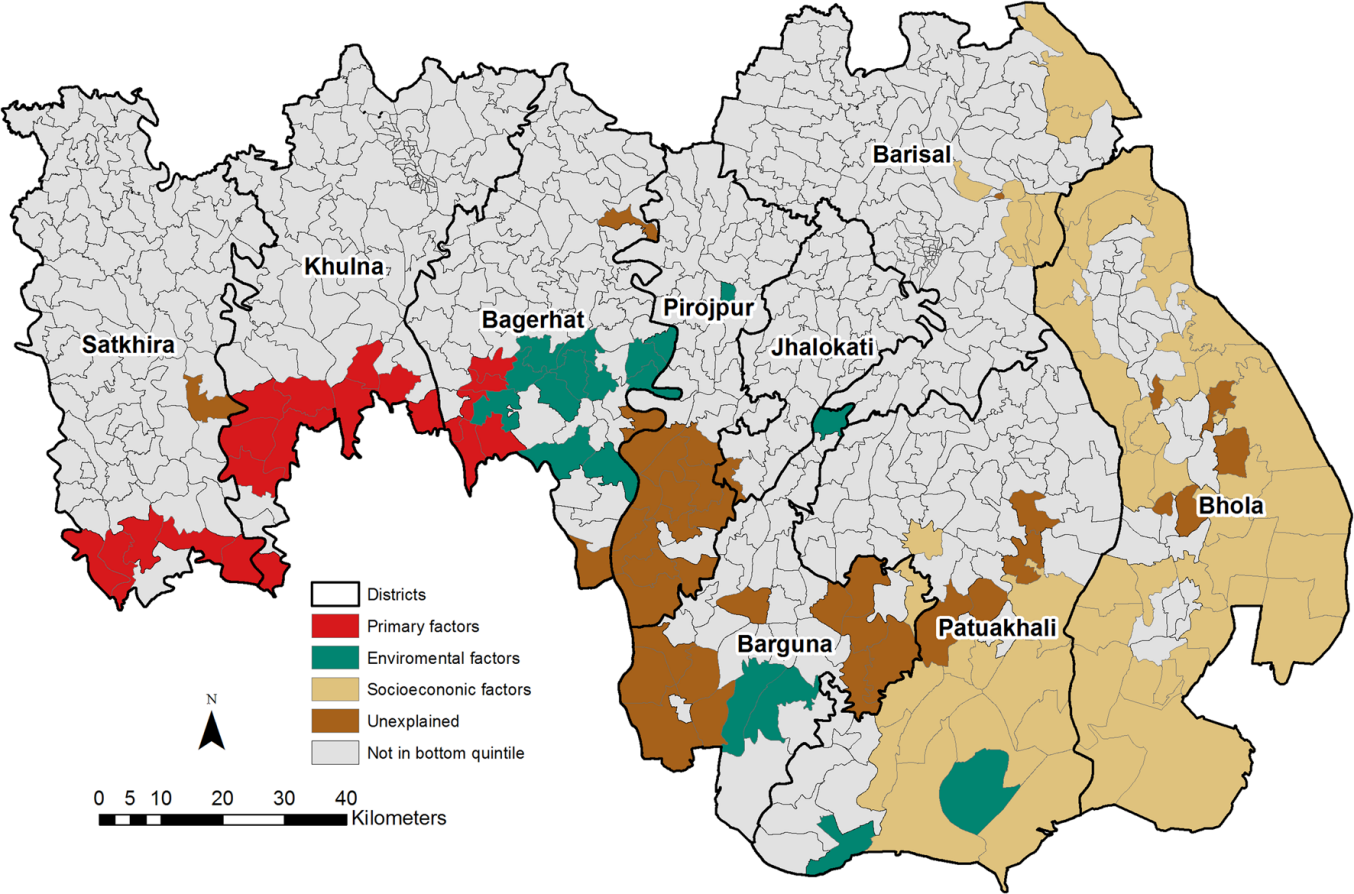
Key drivers of poverty of the unions in the bottom quintile

Figure 4 summarises the spatial correlates of asset poverty for unions in the bottom quintile of the asset score. The figure shows that for unions in the bottom quintile, the primary factors are significantly associated with asset poverty for those in close proximity to the Sundarbans in the Satkhira, Khulna and Bagerhat districts. The environmental controls exhibit significant geospatial associations with asset poverty predominantly with the poorest unions in the Bagerhat (Mithakhali, Baharbunia, Chingrakhali, Hogla Pasha, Khuolia, Nishanbaria, Panchakaran, Putikhali, Ramchandrapur, Teligati, Bhojpatia and Malliker Ber) districts. The environmental controls also exhibited significant associations with poverty in the Pancha Koralia, M.baliatali and Bibichini unions in the Barguna district, Rangabali and Dhulasar unions in the Patuakhali district and also in the Sayna Raghunathpur union in the Pirojpur district.

Figure 4 shows that the socioeconomic factors are important in explaining asset poverty amongst the poorest unions in the Bhola and Patuakhali districts. In the Bhola district, the socioeconomic factors are associated with asset poverty in 34 of the 39 unions in the bottom quintile, constituting 51 % of all the unions in the district. With regards to the Patuakhali district, the socioeconomic factors are associated with asset poverty in 12 of the 18 unions in the bottom quintile. The socioeconomic factors are also important in the Dhulkhola, Hizla Gaurabdi, Alimabad, Char Gopalpur, Jangalia and Bhasan Char unions in the Barisal district and Atharagashia union in the Barguna district.

## Discussions

This study is the first of its kind that examines how environmental stressors (salinity intrusion) and livelihood responses (shrimp farming) are geospatially correlated with poverty in the GBM delta. The Social Safety Net Programmes (SSNP) is the main poverty alleviation strategy for Bangladesh (IMF 2012). The SSNP is primarily aimed at alleviating chronic poverty in Bangladesh and leading to the progress of the country to a middle-income country by 2021 (IMF 2012). Therefore, to ensure that this study is consistent and supports the country’s economic development policies, an asset-based index, which several studies have associated with chronic poverty, was used to identify the poorest unions in the GBM delta. This index is particularly important for developing monitoring strategies, the SSNP are not only conceptualised on short-term goals but more importantly over longer and sustainably goals aimed at enabling the poor to progressively accrue the resources needed to break out of poverty (Cooper and Bird 2012; Stein and Horn 2012).

The findings show a strong clustering of poverty within the GBM coastal delta zone of Bangladesh. More than 50 % of unions in the Bhola and 25 % in the Bagerhat, Barguna, Patuakhali and Pirojpur districts are amongst the poorest unions in the delta. Although the Bagerhat and Pirojpur districts are classified amongst the top four shrimp cultivating districts in the study area (Department of Fisheries 2014; FAO 2015), the findings reveal that poverty remains high in these districts. On the other hand, less than 10 % of unions in the Jhalokati, Barisal, Satkhira and Khulna districts are classified amongst the poorest unions. A comparison between the Union-level outcomes of this study and the Zila and Upazila studies conducted by World Bank et al. (2014) shows that there are clear agreements at the higher levels but at the union level it is clear that Zila and Upazila’s mask high levels of poverty variation at the union level. This becomes clear in Barisal district, where there is a reasonable match of poverty outcomes between the union map presented in this study and the Upazila maps presented by The World Bank et al. (2014). However, the Upazila maps do not capture the extreme poverty around the north of the Sundarbans, confirming the importance of higher resolution poverty maps for policy development.

The geospatial multivariate analysis revealed that after accounting for the significant controls, the levels and intensities of salinity intrusion in a union are significantly associated with an increased probability of a union being in the poorest quintile. However, saline and freshwater shrimp farming are not directly associated with poverty. These findings indicate that despite the asserted monetary benefits of shrimp, its impact on poverty amongst the local populations is trivial. This suggests that shrimp farming in its present structure may not be an effective adaptation to increasing salinity intrusion and poverty, at least not in its current form and not in deltas where shrimp farming might have been thought of as a compensatory livelihood for the loss of agriculture to salinization.

The positive association between salinity intrusion and poverty, primarily due to loss of arable land, agricultural productivity and income, food insecurity, rural unemployment, social unrest, conflicts and forced migration are not disputed in the literature (Sa et al. 2013; Hossain et al. 2013; Swapan and Gavin 2011; Paul and Vogl 2011; Neiland et al. 2001). However, there are contentions on the economic and social benefits of shrimp farming to the poor and marginalised populations in the Bangladeshi delta. Employment opportunities have been cited as the main economic benefit of shrimp farming to the local population in the delta (Islam 2008). Nonetheless, others argue that the marginalisation of the poor, increasing landlessness, collapse of livelihood support systems and the health impacts of shrimp farming exacerbates poverty in the delta (Hossain et al. 2013; Sa et al. 2013; Hagler et al. 2009; Neiland et al. 2001).

The modest benefits of shrimp farming to the local population has been attributed to a number of factors, including industrial scale and more profitable farms being often owned by a few external investors who get the monetary benefits, with the native population only being used as menial labours (Deb 1998; Paul and Vogl 2011). In addition, the shrimp farming sector in Bangladesh is still under-developed. Many local farmers have no formal training in shrimp aquaculture and continue to employ rudimental techniques sated with challenges (Ahsan 2011; Shamsuzzaman and Biswas 2012). Local farmers often encounter problems of low prices, diseases and poor-quality shrimp mostly due to pollution from pesticides and antibiotics limiting their access to the international market (Ahsan 2011). Exploitation by intermediaries within the long distribution chains also deprives farmer of decent profits (Ahsan 2011). These are compounded by violent cyclones and storm surges, particularly Cyclone *Sidr* in 2007 and Cyclone *Aila* in 2009 which led to massive destruction of shrimp ponds driving many of farmers further into poverty (Ahmed and Troell 2010; Rahman et al. 2013). Nonetheless, deforestation for industrial scale shrimp farming has also led to loss of protection from cyclones and storm surges, contributing to decreased coastal defences and increased saline water intrusion (Salam et al. 2003; Ahmed and Troell 2010; Mahmuduzzaman et al. 2014). In this regard, shrimp farming in itself induces salinity and might, therefore, be considered a maladaptation. Perhaps, with the alleviation of these challenges and development of a more sustainable shrimp sector, shrimp farming could potentially become an effective adaption for salinization of the delta.

The findings of the study show that the key drivers of poverty in the delta vary spatially. Whilst salinity intrusion is more important in the poorest unions in the Satkhira and Khulna districts, the environmental factors are important for unions in the Begerhat district. These environmental factors, for example, water logging, pose a direct threat to human wellbeing through the loss of agricultural land and the development of saline soils. The lack of access to markets and other facilities (major roads density) and human capital (education and employment) which could enhance livelihood options and welfare benefits from the findings have a stronger impact in unions in the Patuakhali and Bhola districts.

## Conclusions

Salinity is clearly a driver of poverty in some areas of the delta and it would appear that this might be mediated through both the loss of crops and the changes in livelihood and employment possibly associated in some way with rise of salt water shrimp farming. Shrimp farming remains one of the very few advocated large-scale adaptation options for the rapid salinization of the delta region and poverty reduction. Although shrimp farming has a potential to enhance the wellbeing of the poor and marginalised areas in the delta, this study has shown that shrimp farming has no significant association with poverty. The lack of association might be due to larger, more profitable farms being often owned by external investors (Deb 1998; Paul and Vogl 2011), with less economic benefits to local residents.

This result raises the possibility that at least some saline shrimp farming has predominantly been driven by high profits and only branded as an adaption, whilst failing to address the needs of the poorest in society. It is also possible to conjecture that the reduction in employment associated with shrimp farming may contribute to the well-established migratory behaviours associated with the coastal delta zone, and all the social and urban issues that go with that.

The study further demonstrates that there are a series of drivers of poverty including socioeconomic and environmental factors that are discernible spatially. The Bangladesh Government coastal zone policy identifies that the coastal zone is lagging behind in socioeconomic development and suffers from poor initiatives to cope with different disasters and gradual deterioration of the environment, although it has the potential to contribute much to national development (MoWR 2005). Indeed, drivers of poverty clearly show a spatial pattern and as such policy formulation with regards to social, economic and environmental interventions including addressing landlessness, loss of livelihood support systems could benefit from a more geographical as opposed to purely administrative focus (Hossain et al. 2013). The findings of this study, particularly the spatial differentiation of the main poverty drivers, provide input of relevance to addressing these stated issues in the government’s coastal zone policy.

## Electronic supplementary material

Below is the link to the electronic supplementary material.

Supplementary material 1 (DOCX 32 kb)
