# Supplementary material for: Is shrimp farming a successful adaptation to salinity intrusion? A geospatial associative analysis of poverty in the populous Ganges–Brahmaputra–Meghna Delta of Bangladesh
Source: Sustain Sci. 2016 Mar 21;11(3):423–39. doi: 10.1007/s11625-016-0356-6 (PMC6106650; doi:10.1007/s11625-016-0356-6)
Supplement: Supplementary file 1 — Supplementary material 1 (DOCX 32 kb) [file 11625_2016_356_MOESM1_ESM.docx]

**Appendices**

**Appendix I: Extraction and accuracy assessment of environmental controls**

To calculate union area used for saline and freshwater shrimp farming the Landsat 5TM was radiometric calibrated and atmospherically corrected using the ENVI 5.0 FLAASH tool set to the Tropical Atmospheric Model (Exelis Visual Information Solutions 2012). Subsequent processing was run in ESRI ArcGIS 10.2 (ESRI 2013). The scenes were mosaicked into a single seamless image and a composite image using bands 4, 5 and 3 was constructed. This band combination offers better definition for water courses, wetter soils appear darker and vegetation as variations in brown and orange colours. Using the ISOLCLUSTER tool for unsupervised classification, the composite image was classified into 10 classes, capturing the dominant land covers within the study region. This raster was clipped back to the study area and passed through a majority filter which replaces cells based on the majority value in their contiguous neighbourhoods. This smooths the raster reducing the spectral noise created by unsupervised classification. A separate raster was built from the seamless image using the Normalised Water Difference index (McFeeters 1996) and water pixels vectorised. A visual inspection of this data was carried out to identify what was clearly river channel from flooded fields.

The river channels identified from the visual inspection were converted back into a raster and mosaicked with the unsupervised classification raster. This improved the delineation of the river network within the study area and it’s separation from aquaculture or water-logged land cover classes. The 2010 limits of the cities of Khulna and Barisal were digitised and merged into the land use raster. A Mangrove mask was manually delineated and used to reclassify spurious pixel values primarily within the Sundarban regions. The remaining pixel values identified areas of bare ground or rural settlement. The final processing step aggregated pixel values considered to represent a single class into a single value. The final raster identified 9 land use classes (Fig. 1).

Aquaculture pixels were reclassified into fresh and saline water shrimp farms using salinity regime and the associated structure of aquaculture ponds. Aquaculture pixels near the Sundarbans and coast are considered as saline water shrimp farms as the area comes under high saline regime whereas the aquaculture pixels more inner part of the land where the soil and ground water salinity is less or nil were taken as fresh water farms. Moreover fresh water aquaculture farms are typically smaller in shape with more vegetation cover than saline aquaculture farms. These differences were used to reclassify aquaculture pixels into fresh or saline aquaculture farms using post classification manual editing using comparatively 24 meter resolution IRS P6 (ResourceSat-1) and IRS R2 (Resourcesat-2) provided by the Indian Space Research Organization. This was used to calculate the percentage of union area used for fresh and saline water shrimp farms.

To identify water logged agricultural lands, the Landsat was augmented with surface soil moisture content from the MODIS TSI. A Temperature-Vegetation Dryness Index proposed by Sandholt et al. (2002) was used to derive surface soil moisture from the MODIS TSI for March, April and May 2010. The dry season (November to March; cool, dry winter) in Bangladesh is followed by a pre-monsoon season (March to June; hot, humid summer) with gradually increasing precipitation. Irrigation only occurs in the dry season in coastal Bangladesh; therefore, observed high soil moisture in the March, April and May generally implies soils that have poor drainage properties and thus can be susceptible to water logging caused by both rain and flooding events in any part of the year. Based on this information, we calculated the percentage of water logged agricultural land in each union. Ahsan (2012) documented that moderate to very high salinity intrusion can lead to prolonged artificial water logging with saline water for aquaculture and high existence of toxic buried acid sulphate layer with adverse effects for agriculture productivity.

An accuracy assessment was conducted on the 2010 land cover map. The project lacked the resources to ground truth sites within the study region, thus the assessment was conducted using photo interpretation of Google Earth imagery from the same period. Stratification by the land cover classes were used to ensure that all classes were sampled. Seventy-five random locations per class were selected based on the recommendations of Lillesand and Kiefer (2000). Sampling points were converted to polygons (30x30m), the resolution of the Landsat and exported into KML so they could be displayed in Google Earth. The assessment was conducted by an individual with substantial years of experience visiting and working in the region.

Table 1 show the error matrix of the 675 sampled units by class. The table shows that the majority of water logged agricultural land has been assigned to non-water logged agricultural land. The reason being that water-logged agricultural land is a class identified using surface soil moisture content from MODIS an attribute that is not visible within Google Earth imagery. Therefore, the assessor had no means of identifying water-logged agricultural land. In this regard, the two agricultural land classes were aggregated into a single class – agricultural land (Table 2). From Table 2, the proportion of correctly classified land cover is 80% (Overall Accuracy – OA). The Kappa statistics (76%) which compares the observed accuracy with the expected accuracy (random chance) shows that there is almost a perfect agreement between what was observed and what was expected (Munoz and Bangdiwala 1997).

The User Accuracy is the probability of a pixel on the map representing the correct land cover category. In other words from the perspective of the assessor how accurate was the map. Mangrove, agricultural land and saline water shrimp farms had the highest user accuracy (>90%). Major urban area has a user accuracy of 80% with the bulk of the 20% commission error identified as rural settlement. The definition of Major urban area was defined by the urban limits of 2 key cities within the study region and it is clear that within these urban limits is a patch work of dense urban area and rural settlement like habitats. Rural settlement has a user accuracy of 73% with the bulk of the commission error identified as agriculture. This is unsurprising as rural settlement is set within a landscape of predominantly agricultural areas. Twenty-one percent of freshwater shrimp farms are identified as saline shrimp farms. A key separation of these two shrimp farms is their shape and size, a property that in some cases may be too small for Landsat, yet clearly visible in Google Earth. Wetland/Mudflats were poorly identified; the principal source of error is that these narrow linear features are missed through spatial misalignment with Google Earth imagery.

Producer Accuracy is the probability of reference pixel being correctly classified. In other words from the perspective of the map maker how accurate was the classification? Major urban area, mangrove and freshwater shrimp farms all had high procedure accuracy (>= 90%). Rural settlement, agriculture and wetland/mudflats had similar but lower producer accuracy. Saline shrimp farms had the lowest producer accuracy and it is clear the bulk of the omission errors were identified as freshwater shrimp farms, these are likely due to misclassification of shrimp farms with similar size.

**References**

ESRI (2013) ArcGIS 10.2. Environmental Systems Resource Institute, Redlands, California.

Exelis Visual Information Solutions (Ed.), 2012. ENVI Documentation, ENVI v5.0.

Lillesand TM, Kiefer RW (2000) Remote Sensing and Image Interpretation, 4th ed., John Wiley: New York.

McFeeters SK (1996) The use of normalized difference water index (NDWI) in the delineation of open water features. International Journal of Remote Sensing 17:1425-1432.

Munoz SR, Bangdiwala SI (1997) Interpretation of Kappa and B statistics measures of agreement. Journal of Applied Statistics 24(1):105-112, doi: 10.1080/02664769723918.

Sandholt I, Rasmussen K, Andersen J (2002) A simple interpretation of the surface temperature/vegetation index space for assessment of surface moisture status. Remote Sensing of Environment 79(2-3):213-224.

Table 1: Error matrix sampled units by class

| Map Class | Reference data –observed using Google Earth | | | | | | | | | Total |
| --- | --- | --- | --- | --- | --- | --- | --- | --- | --- | --- |
|  | Non-water logged agricultural land | Water logged Agricultural land | Saline water shrimp farm | Freshwater shrimp farm | Major Urban Area | Mangrove | Rural Settlement | Water | Wetland/Mudflats |  |
| Non-water logged agricultural land | 68 | 0 | 2 | 0 | 0 | 0 | 1 | 3 | 1 | 75 |
| Water logged agricultural land | 71 | 0 | 0 | 0 | 0 | 0 | 4 | 0 | 0 | 75 |
| Saline water shrimp farm | 2 | 2 | 68 | 1 | 0 | 0 | 0 | 1 | 1 | 75 |
| Freshwater shrimp farm | 3 | 0 | 16 | 55 | 0 | 0 | 0 | 1 | 0 | 75 |
| Major Urban Area | 0 | 0 | 0 | 2 | 60 | 0 | 13 | 0 | 0 | 75 |
| Mangrove | 0 | 0 | 0 | 0 | 0 | 75 | 0 | 0 | 0 | 75 |
| Rural Settlement | 15 | 0 | 2 | 0 | 0 | 1 | 55 | 2 | 0 | 75 |
| Water | 0 | 0 | 5 | 2 | 0 | 1 | 1 | 62 | 4 | 75 |
| Wetland/Mudflats | 30 | 1 | 8 | 1 | 0 | 2 | 3 | 7 | 23 | 75 |
| Total | 189 | 3 | 101 | 61 | 60 | 79 | 77 | 76 | 29 | 675 |

Table 2: Error matrix sampled units by class – non-water logged agricultural land and water logged agricultural land combined)

| Map Class | Reference data –observed using Google Earth | | | | | | | | Total | User  Accuracy (%) | Commission Error (%) |
| --- | --- | --- | --- | --- | --- | --- | --- | --- | --- | --- | --- |
|  | Agricultural land | Saline water shrimp farm | Freshwater shrimp farm | Major Urban Area | Mangrove | Rural Settlement | Water | Wetland/Mudflats |  |  |  |
| Agriculture | 139 | 2 | 0 | 0 | 0 | 5 | 3 | 1 | 150 | 93 | 7 |
| Saline water shrimp farm | 4 | 68 | 1 | 0 | 0 | 0 | 1 | 1 | 75 | 91 | 9 |
| Freshwater shrimp farm | 3 | 16 | 55 | 0 | 0 | 0 | 1 | 0 | 75 | 73 | 27 |
| Major Urban Area | 0 | 0 | 2 | 60 | 0 | 13 | 0 | 0 | 75 | 80 | 20 |
| Mangrove | 0 | 0 | 0 | 0 | 75 | 0 | 0 | 0 | 75 | 100 | 0 |
| Rural Settlement | 15 | 2 | 0 | 0 | 1 | 55 | 2 | 0 | 75 | 73 | 27 |
| Water | 0 | 5 | 2 | 0 | 1 | 1 | 62 | 4 | 75 | 83 | 17 |
| Wetland/  Mudflats | 31 | 8 | 1 | 0 | 2 | 3 | 7 | 23 | 75 | 31 | 69 |
| Total | 192 | 101 | 61 | 60 | 79 | 77 | 76 | 29 | 675 |  |  |
| Producer Accuracy (%) | 72 | 67 | 90 | 100 | 95 | 71 | 82 | 79 |  | OA | 80 |
| Omission Error (%) | 28 | 33 | 10 | 0 | 5 | 29 | 18 | 21 |  | Kappa | 76 |

**Appendix II: Statistical analysis**

This section describes the Bayesian Geo-additive Semi-parametric regression procedure used for the multivariate spatial analysis. The outcome variable $y_{i}$ is coded 1 if a union is in the asset poverty bottom quintile and 0 otherwise. In this regard, the outcome variable $y_{i}$ follows a binomial distribution with parameters $n_{i}$ and $\pi_{i}$; that is $y_{i}\sim B(n_{i}, \pi_{i})$, where $\pi_{i}$ is the probability of a union being in the bottom quintile and $n_{i}$ is the number of unions in the Delta. The model linking the probabilities of a union being in the bottom asset wealth quintile with the covariates is the logistic model of the form

$\pi_{i}=P(y_{i}1|\eta_{i)}=\frac{exp(\eta_{i})}{1+exp(\eta_{i})}$ 1

where $\eta_{i}$ is the predictor of interest. If we have a vector $x_{i}^{'}={(x_{i1},\ldots,x_{ik})}^{'}$ of *k* continuous covariates and $\lambda_{i}^{'}={(\lambda_{i1},\ldots\lambda_{id})}^{'}$ a vector of *d* categorical covariates, then predictor $\eta_{i}$ can be specified as

$\eta_{i}=\alpha\lambda_{i}^{'}+\beta x_{i}^{'}$ 2

Where $\alpha$ is a *d*-dimensional vector of unknown regression coefficients for the categorical covariates $\lambda_{i}^{'}$ ,$\beta$ is a *k*-dimensional vector of unknown regression coefficients for the continuous covariates $x_{i}^{'}$ . To account for non-linear effects of the continuous covariates and spatial dependence in asset wealth, the BGS framework which replaces the strictly linear predictors with flexible semi-parametric predictors was adopted. The BGS model is then specified as shown in Equation 3 below

$\eta_{i}=\alpha\lambda_{i}^{'}+f_{k}x_{ik}^{'}+f^{spat(S_{i})}$ 3

where $f_{k}(x)$ are non-linear smoothing function of the continuous variables $x_{ik}$ and $f^{spat(S_{i})}$ accounts for unobserved spatial heterogeneity at location *i* (*i*=1,...,S), some of which may be spatially structured and others unstructured. The spatially structured effects shows the effect of location by assuming that geographically close areas are more similar than distant areas, whist the unstructured spatial effect accounts for spatial randomness in the model. In this regard, equation 3 can be specified as

$\eta_{i}=\alpha\lambda_{i}^{'}+f_{k}x_{ik}^{'}+f^{str(S_{i})}+f^{unstr(S_{i})}$ 4

where $f^{str}$ is the structured spatial effects and $f^{unstr}$ is the unstructured spatial effects and $f^{spat(S_{i})}=f^{str}+f^{unstr}$. In the case of this study, the spatially structured effects depicts the extent of clustering of asset poverty and the influence of unaccounted predictor variables that themselves may be spatially clustered ‎or random. The smooth effects of continuous factors are modelled with P-spline priors, whilst the spatial effects are modelled using Markov random field priors. BGS models produce a map of the posterior spatial effect, which allows for the impact of the covariates in explaining the spatial patterns of poverty to be examined.
